# Toronto Veterinary College

**Published:** 1885-04

**Authors:** 


The Toronto Veterinary College.—The January number of the Journal contains an editorial entitled "Veterinary Questions in the United States," in which we inadvertently ignored the claim of the above named college to be worthy of public patronage. The college in question has well sustained its standard of excellence, and if we object to its two years' course, deeming a longer term of the utmost importance, we do not pretend to condemn the institution itself, as the same objection could be raised against others of high standing.

